# Impact of Interstitial Ni on the Thermoelectric Properties of the Half-Heusler TiNiSn

**DOI:** 10.3390/ma11040536

**Published:** 2018-03-30

**Authors:** Sonia A. Barczak, Jim Buckman, Ronald I. Smith, Annabelle R. Baker, Eric Don, Ian Forbes, Jan-Willem G. Bos

**Affiliations:** 1 Institute of Chemical Sciences and Centre for Advanced Energy Storage and Recovery, School of Engineering and Physical Sciences, Heriot-Watt University, Edinburgh EH14 4AS, UK; sb306@hw.ac.uk; 2 Institute of Petroleum Engineering, Heriot-Watt University, Edinburgh EH14 4AS, UK; J.Buckman@hw.ac.uk; 3 ISIS Facility, Rutherford Appleton Laboratory, Harwell Campus, Didcot OX11 0QX, UK; ron.smith@stfc.ac.uk; 4 Diamond Light Source, Harwell Campus, Didcot OX11 0DE, UK; annabelle.baker@diamond.ac.uk; 5 SemiMetrics Ltd., Kings Langley WD4 9WB, UK; eric.don@semimetrics.com; 6 Department of Physics and Engineering, Northumbria University, Newcastle NE1 8ST, UK; ian.forbes@northumbria.ac.uk

**Keywords:** half-Heusler, TiNiSn, thermal conductivity, thermoelectric materials

## Abstract

TiNiSn is an intensively studied half-Heusler alloy that shows great potential for waste heat recovery. Here, we report on the structures and thermoelectric properties of a series of metal-rich TiNi_1+y_Sn compositions prepared via solid-state reactions and hot pressing. A general relation between the amount of interstitial Ni and lattice parameter is determined from neutron powder diffraction. High-resolution synchrotron X-ray powder diffraction reveals the occurrence of strain broadening upon hot pressing, which is attributed to the metastable arrangement of interstitial Ni. Hall measurements confirm that interstitial Ni causes weak n-type doping and a reduction in carrier mobility, which limits the power factor to 2.5–3 mW m^−1^ K^−2^ for these samples. The thermal conductivity was modelled within the Callaway approximation and is quantitively linked to the amount of interstitial Ni, resulting in a predicted value of 12.7 W m^−1^ K^−1^ at 323 K for stoichiometric TiNiSn. Interstitial Ni leads to a reduction of the thermal band gap and moves the peak ZT = 0.4 to lower temperatures, thus offering the possibility to engineer a broad ZT plateau. This work adds further insight into the impact of small amounts of interstitial Ni on the thermal and electrical transport of TiNiSn.

## 1. Introduction

The conversion of waste heat into electricity using thermoelectric generators is a possible route to reduce reliance on fossil fuels and reduce our carbon footprint [1,2]. However, modest conversion efficiencies and relatively high costs limit large scale applications [3,4]. The efficiency of a thermoelectric material is determined by its figure of merit:

(1) ZT = (S^2^/κρ)T

Efficient, high-ZT materials must simultaneously have a high Seebeck coefficient (S), low electrical resistivity (ρ), and low thermal conductivity (κ), where κ is the sum of a lattice (κ_lat_) and an electronic (κ_el_) contribution (T is the absolute temperature). This is a challenging problem due to the interdependence of the thermoelectric parameters, leading to the identification of heavily doped semiconductors as the most promising thermoelectric materials [5].

Half-Heusler (HH) alloys are important thermoelectric materials for mid-temperature waste heat recovery. The best compositions are based on ZrNiSn (n-type), ZrCoSb (p-type) or NbFeSb (p-type) and have ZT = 1–1.5 at 800–1100 K [6,7,8,9,10]. Recently, NbCoSb compositions with Nb vacancies have been reported to have ZT approaching unity, providing a new addition to this class of thermoelectric materials [11,12,13]. In terms of their thermoelectric parameters, HHs are characterised by large power factors S^2^/ρ = 4–6 mW m^−1^ K^−2^ but are limited by a large κ_lat_ = 3–4 W m^−1^ K^−1^ for optimised compositions [14,15]. Besides promising ZT values, HH alloys have good mechanical properties, good thermal stability and are composed of relatively cheap elements, if the use of Hf can be avoided. For this reason, TiNiSn based HHs have attracted significant interest. One possible route to reduce κ_lat_ is the introduction of excess Ni, yielding ZT = 0.5–0.7 in TiNi_1+y_Sn HH alloys (y = 0.05–0.15) [16,17,18,19]. It is well-known that TiNiSn and the full-Heusler (FH) TiNi_2_Sn do not form a full solid solution and segregate into HH and FH phases beyond a small solubility limit [20,21]. Our experimental work and that of others indicates that up to ~8% excess Ni can be accommodated prior to the observation of FH phases in diffraction patterns [18,19,22,23,24,25]. Microscopy studies confirm the segregation into HH and FH phases with the arrangement of the excess Ni within the HH phase dependent on sample processing [16,17,19,26,27,28,29]. Our work on samples prepared using solid-state reactions indicates that most of the excess metals are present as randomly distributed interstitials with no evidence for large numbers of nanometer sized FH inclusions [23,30]. The tendency towards phase segregation is instead manifested by grain by grain compositional variations and the presence of wetting layers at grain boundaries after hot pressing [30]. Exploiting Cu interstitials in TiNiSn leads to efficient n-type doping and rapid reduction of κ_lat_, resulting in viable thermoelectric performance in a HH alloy based on abundant elements [30].

Here, we investigate the thermoelectric properties of TiNi_1+y_Sn HH alloys prepared using solid-state reactions and hot pressing. We present the results of Hall measurements and Callaway modelling and show that interstitial Ni strongly influences both the electrical and thermal transport.

## 2. Materials and Methods

Polycrystalline TiNi_1+y_Sn (y = 0, 0.02, 0.075, 0.25) and TiNiSn with 2% excess Ti samples were synthesised on a 5-gm scale via solid-state reaction of high purity elemental powder precursors (Alfa Aesar, Heysham, UK; Ti, 325 mesh; Ni, 120 mesh; Sn, 100 mesh; ≥99.8% purity). The sample with excess Ti was prepared to check if it is possible to reduce the amount of spontaneous excess Ni that is found in TiNiSn-based HH alloys [23,24]. The starting materials were thoroughly mixed using an agate mortar and pestle and cold pressed using a 10-ton press into 13 mm diameter pellets (pressure 600–700 MPa). Samples were wrapped in 0.025 mm thick Ta foil (Alfa Aesar) to prevent surface oxidation and initially annealed in evacuated quartz tubes at 850 °C for 24 h using 10 °C/min heating and 20 °C/min cooling stages. The mixtures were then re-ground, cold pressed, wrapped in Ta foil, and heated in vacuum sealed quartz tubes for a further two weeks at 850 °C to ensure a complete reaction. For this second step, the samples were inserted directly into the furnace at 850 °C and at the end of the two-week heating period they were quenched from 850 °C. The resulting products were ground using a mortar and pestle and hot pressed into 13 mm diameter disks of ~2 mm thickness at 950 °C and 80 MPa for 20 min using a home-built system. Densities were determined using the Archimedes method and were found to be 90–95% of the theoretical density (Table 1).

The structure and purity of the prepared materials were investigated by laboratory X-ray powder diffraction (XRD) using monochromated CuKα_1_ radiation (Bruker, D8 Advance; Billerica, MA, USA). 8-h scans collected over 10° ≤ 2θ ≤ 120° on finely ground samples were used for initial Rietveld refinements. Neutron powder diffraction (NPD) data were collected at room temperature from 1–2 g of finely ground hot-pressed samples using the Polaris instrument at the ISIS neutron and muon source, Rutherford Appleton Laboratory, Didcot, UK. Rietveld refinement using both XRD and NPD data were performed with the General Structure Analysis System (GSAS) and its graphical user interface, EXPGUI [31,32]. Synchrotron X-ray diffraction (SXRD) data (λ = 0.825921 Å, step size 0.002°) were collected from a subset of the samples on the high-resolution powder diffraction beamline I11 at the Diamond Light Source, Didcot, UK. The collection time for samples prior to densification was 30 min while, for the hot-pressed samples, 1-h data collection time was used. Rietveld refinements using the SXRD data were performed with the TOPAS Academic software package. The microstructure and homogeneity of the samples were confirmed using a Quanta 650 FEG Scanning Electron Microscope (FEI, Eindhoven, The Netherlands) operated at 20 kV in high-vacuum and equipped with an Oxford Instruments X-max^N^ 150 mm detector for energy dispersive X-ray (EDX) mapping. The EDX mapping was performed without further calibration. Prior to analysis, the surface of each sample was polished with fine Al_2_O_3_ sandpaper down to 0.3 µm roughness.

For the electrical property measurements, rectangular bars were cut using a low-speed saw with a diamond blade. The electrical resistivity (ρ) and Seebeck coefficient (S) were measured under a He atmosphere using a Linseis LSR-3 instrument (Linseis, Selb, Germany). Hall measurements were taken using the method of van der Pauw using a magnetic field ±1 T, DC current ≤±100 mA leading to Hall voltages ±0.1–10 mV measured using a nanovoltmeter [33]. Silver paint was used to make contacts on the corners of ~5 × 5 × 1 mm^3^ disks, which were clamped into a probe card for measurement. The thermal diffusivity (α) was measured under vacuum between RT and 773 K using a Linseis LFA-1000 instrument (Linseis, Selb, Germany). To minimise errors in the emissivity, the disks were coated using a carbon spray. The total thermal conductivity (κ) was calculated according to the formula κ = αdC_p_, where d is the sample density and C_p_ is the heat capacity. The heat capacity of TiNiSn was used for all samples and is published in [30]. A porosity correction was applied to the calculated κ values: κ/κ_dense_ = 1 − (4/3)ϕ; ϕ = (100 − %density)/100.

## 3. Results

### 3.1. X-ray Powder Diffraction

**Figure 1:**
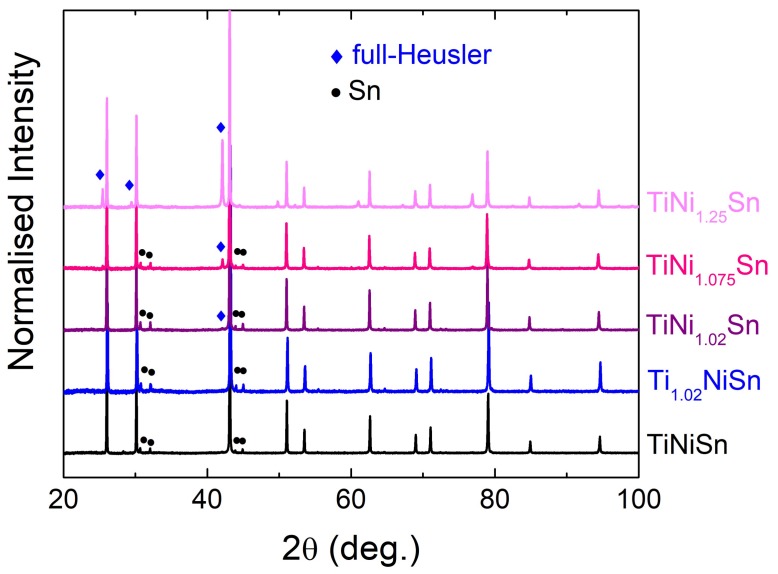
Laboratory X-ray (Cu K_α1_) powder diffraction patterns for the TiNi_1+y_Sn series and Ti_1.02_NiSn. Data are normalised and offset along the intensity axis and plotted against 2θ angle.

Laboratory XRD data from the TiNi_1+y_Sn series and the Ti_1.02_NiSn sample after hot pressing are shown in Figure 1. In addition to the main HH reflections, a small Sn impurity is apparent in most samples. The y = 0 and Ti_1.02_NiSn samples do not contain any FH phase, while the y = 0.02 and 0.075 samples have a small amount of FH present. The y = 0.25 sample is a clear two-phase mixture of (Ni-rich) HH and FH phases. Rietveld analysis was used to obtain accurate lattice parameters and weight fractions of the phases present. These are summarised in Table 1.

### 3.2. Neutron Powder Diffraction

**Figure 2:**
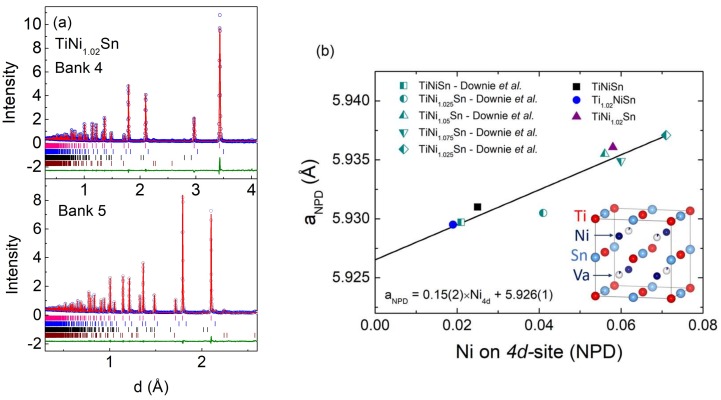
(**a**) Observed (blue circles), calculated (red line) and difference (green line) Rietveld neutron powder diffraction profiles for Ti_1.02_NiS. Bragg reflection markers correspond to (from top to bottom): half-Heusler; Sn and Ti. Banks 4 and 5 are the 90° and backscatter (2θ ~ 147°) detector banks on the Polaris instrument. (**b**) Interstitial Ni_4d_ site occupancy dependence of the half-Heusler lattice parameter using literature data [23,30] and the samples discussed here. The solid line is a linear fit.

NPD data were collected from the Ti_1.02_NiSn and TiNi_1.02_Sn samples, while data from the TiNiSn sample was reported in [30]. The main purpose of this analysis was to obtain the experimental site occupancies of the HH phase. The Ti, Ni, and Sn atoms occupy the 4a (Ti), 4c (Ni), and 4b (Sn) positions, while any excess Ni is accommodated on the 4d position. The results of these fits are summarised in Table 2 and the final fit to the TiNi_1.02_Sn sample is shown in Figure 2a. Rietveld analysis yields compositions of Ti_0.978(3)_Ni_1.019(1)_Sn (Ti_1.02_NiSn) and Ti_0.977(2)_Ni_1.058(1)_Sn (TiNi_1.02_Sn). The nominally stoichiometric TiNiSn sample has a fitted Ti_0.974(3)_Ni_1.025(1)_Sn composition. This analysis therefore confirms that TiNiSn based compositions tend to form with a small amount of spontaneous excess Ni beyond that expected based on the starting composition [23,24]. This contrasts with TiCoSb where no evidence for excess Co was found from NPD [34]. The Ti_1.02_NiSn sample has the lowest amount of interstitial Ni, suggesting that the additional Ti helps to reduce the amount of excess Ni. However, NPD data reveals the presence of unreacted elemental Ti in all samples, suggesting that the spontaneous excess Ni is caused by an incomplete reaction, rather than loss of Ti due to for example oxidation.

Figure 2b shows the dependence of the HH lattice parameter on the fitted amount of Ni on the Ni_4d_ interstitial site. This graph combines previously published data [23,30] and the two samples reported here. The lattice parameter shows a linear dependence on the amount of interstitial Ni with the following relation: a_HH_ (Å) = 0.15(2) × Ni_4d_ + 5.926(1). This enables an estimate of the amount of excess Ni within the HH phase for the TiNi_1+y_Sn samples for which no NPD data is available. The experimental Ni_4d_ occupancies are 0.08(1) (y = 0.075) and 0.09(1) (y = 0.25), in agreement with the expected solubility limit. A full overview of the amount of interstitial Ni in the HH phase is given in Table 3. The linear relationship can also be used to predict that stoichiometric TiNiSn should have a lattice parameter of 5.926(1) Å.

### 3.3. Synchrotron X-ray Powder Diffraction

**Figure 3:**
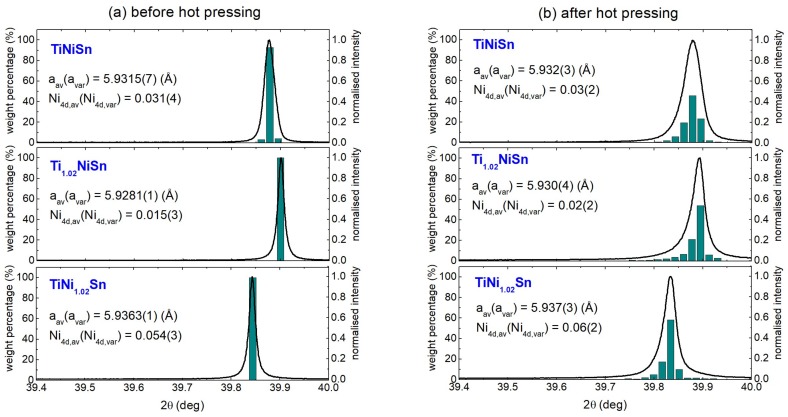
Close-up of the (422) reflection in synchrotron X-ray powder diffraction for the TiNiSn, Ti_1.02_NiSn, and TiNi_1.02_Sn samples (**a**) before and (**b**) after hot pressing. The green histograms indicate the HH phases used to fit the peak shape. The average lattice parameter (a_av_), peak broadening (a_var_), average Ni_4d_ occupancy (Ni_4d,av_), and variance of the Ni_4d_ occupancy (Ni_4d,var_) are shown as a_av_(a_var_) and Ni_4d,av_(Ni_4d,var_) in the panels. Peak intensities have been normalised and data are plotted against 2θ angle.

SXRD data were collected from the TiNiSn, Ti_1.02_NiSn, and TiNi_1.02_Sn samples before and after hot pressing to estimate the impact of the combined pressure and high-temperature consolidation step. As shown in Figure 3, the Bragg peaks of compositions prior to hot pressing are highly symmetric suggesting homogeneous, strain-free materials. By contrast, the Bragg reflections of all samples after hot pressing show asymmetry and strain broadening towards higher d-spacing. This behaviour was also observed for the TiNiCu_y_Sn samples and is caused by segregation of interstitial metals [30]. We modelled the peak broadening using a set of HH phases with small increments in lattice parameters (∆a = 0.0025 Å) and profile parameters obtained from the symmetric shape prior to hot pressing. Using this approach [29], it is possible to obtain a quantitative histogram of lattice parameter and abundance for the HH phases present (see Figure 3). All samples before hot pressing are fitted well using a single phase with highly symmetric peak shape. After hot pressing, the reflections are strongly broadened and ≥3 significant HH phases are needed to describe the peak shape. Inspection reveals that a small part of the sample has an increased lattice parameter, while the majority has a somewhat reduced lattice parameter, with a nearly constant average.

To quantify the change in average lattice parameter (a_av_) and peak width (a_var_) we define the following functions for the average:

(2) aav=∑iwiai∑iwi

and variance:

(3) avar2=∑iwi(ai−aav)2∑iwi

Here, w_i_ and a_i_ are the weights and lattice parameters of the HH phases used to fit the SXRD data. The linear relationship between Ni_4d_ occupancy and HH lattice parameter (Figure 2b) allows a_av_ and a_var_ to be converted into an average site occupancy and compositional variation, respectively. The resulting values are given as a_av_(a_var_) and Ni_4d,av_(Ni_4d,var_) in the panels of Figure 3. This reveals that the average composition of the HH phase does not change significantly upon hot pressing. By contrast, the variance of the Ni_4d_ occupancy increases significantly from ±0.003 before hot pressing to ±0.02 after hot pressing. This demonstrates that hot pressing leads to significant changes in the distribution of the interstitial Ni, which is driven by the tendency towards formation of either HH or FH grains and extrusion of excess metals towards grain boundaries [30].

### 3.4. Scanning Electron Microscopy

**Figure 4:**
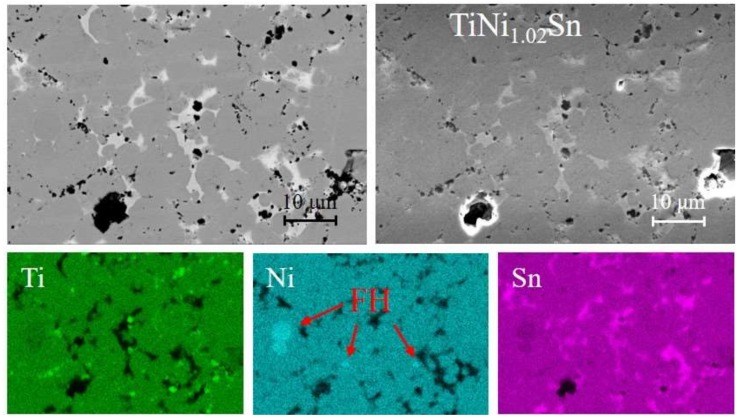
Backscattered electron (BSE, left) and secondary electron (SE, right) SEM images and EDX maps (Ti, Ni, and Sn respectively) for TiNi_1.02_Sn. The elemental maps cover the same area as the BSE and SE images.

SEM-EDX was used to investigate the TiNi_1.02_Sn sample, which is representative of the samples studied here. The backscattered electron (BSE) image, secondary electron (SE) image, and EDX maps are shown in Figure 4. Significant amounts of elemental Sn are evident, in agreement with the Rietveld analysis that reveals ~4 wt % elemental Sn (Table 1). The EDX analysis also confirms the presence of elemental Ti in these samples. Several small FH regions (~1–5 µm^2^) corresponding to TiNi_2_Sn were observed, in keeping with the observation of this phase in XRD and NPD. In terms of the microstructure, the sample is well sintered with large average grain sizes with µm scale pores responsible for the non-ideal 95(2)% density for this sample (Table 1).

### 3.5. Thermoelectric Properties

**Figure 5:**
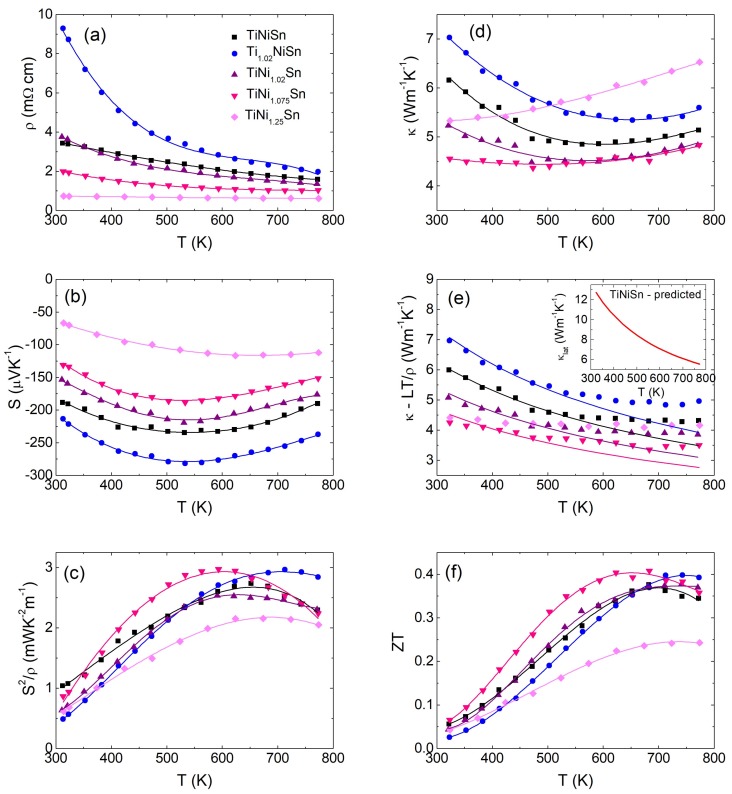
Temperature dependence of (**a**) the electrical resistivity (ρ), (**b**) the Seebeck coefficient (S), (**c**) the thermoelectric power factor (S^2^/ρ), (**d**) the total thermal conductivity (κ), (**e**) the lattice thermal conductivity (κ-LT/ρ) and (**f**) the figure of merit (ZT) for the TiNi_1+y_Sn samples and Ti_1.02_NiSn. The solid lines in panel (e) are Debye–Callaway fits in the 323–523 K interval. The inset to panel (e) shows the predicted κ_lat_ for stoichiometric TiNiSn.

The temperature dependence of S, ρ, the power factor, S^2^/ρ, the total (κ) and lattice thermal (κ-LT/ρ) conductivities and ZT are shown in Figure 5. The ρ(T) and S(T) for the TiNi_1+y_Sn (y ≤ 0.075) samples and Ti_1.02_NiSn are typical of non-degenerate semiconductors with a maximum in S(T) near 500 K and a thermally activated semiconducting ρ(T) (Figure 5a,b). The maximum in S(T) is caused by the excitation of minority p-type charge carriers, leading to a decrease in S. The presence of minority carriers at elevated temperatures is confirmed by the observation of a bipolar electronic contribution to κ(T) above ~500 K (Figure 5d,e). The magnitude of S scales with the amount of interstitial Ni, with a decrease from −210 µV K^−1^ (Ni_4d_ = 0.019) to −190 µV K^−1^ (Ni_4d_ = 0.025) to −150 µV K^−1^ (Ni_4d_ = 0.058) to −125 µV K^−1^ (Ni_4d_ = 0.08) at 313 K. The impact of the interstitial Ni is therefore consistent with gradual n-type doping that reduces the magnitude of S but maintains the semiconducting S(T). The magnitude of ρ follows the Ni_4d_ site occupancy and reduces from 9 mΩ cm (Ni_4d_ = 0.019) to 2 mΩ cm (Ni_4d_ = 0.08). S^2^/ρ increases from 0.5–1 mW m^−1^ K^−2^ at 313 K to maximum values of 2.5–3 mW m^−1^ K^−2^ at 550–700 K. These values are comparable to other literature reports [16,19,25], although in some studies larger S^2^/ρ ≥ 4 mW m^−1^ K^−2^ have been reported [18,35]. The y = 0.25 sample contains 26 wt % metallic FH phase and this sample should be considered a composite of HH (Ni_4d_ = 0.09) and FH phases. S(T) shows much less temperature dependence and is characterised by a broad maximum, while ρ(T) is almost temperature independent, tending towards metal-like behaviour. S^2^/ρ is reduced with (S^2^/ρ)_max_ = 2.2 mW m^−1^ K^−2^ at 675 K. From the perspective of obtaining large S^2^/ρ, the presence of large amounts of metallic FH phases therefore does not appear to be favourable.

κ(T) and κ-LT/ρ are shown in Figure 5d,e. Here, the temperature dependent Lorenz number was evaluated following [36]. As already indicated, κ(T) and κ-LT/ρ show clear evidence for a bipolar contribution above 500–550 K. Subtraction of the Wiedemann–Franz term (LT/ρ) therefore yields κ_lat_ below ~550 K. All samples (except the y = 0.25 HH/FH composite) follow a T^−z^ temperature dependence between 323–500 K. Log–log plots reveal that the exponent z decreases from 0.56(3) (Ni_4d_ = 0.019) to 0.30(2) (Ni_4d_ = 0.08). A slope z = 1 is indicative of thermal transport limited by Umklapp scattering, while z = 0.5 is expected for point-defect phonon scattering [15]. The data therefore indicate increased point-defect phonon scattering and reduced κ_lat_ as the amount of interstitial Ni increases. The favourable impact is also evident from the decrease of κ_lat, 323K_ = 7 W m^−1^ K^−1^ (Ni_4d_ = 0.019) to κ_lat, 323K_ = 6.0 W m^−1^ K^−1^ (Ni_4d_ = 0.025) to κ_lat, 323K_ = 5.1 W m^−1^ K^−1^ (Ni_4d_ = 0.058) to κ_lat, 323K_ = 4.2 W m^−1^ K^−1^ (Ni_4d_ = 0.08) as the amount of interstitial Ni increases. κ(T) for the y = 0.25 HH/FH composite sample increases with temperature. Evaluation of the lattice component is complicated by the two-phase nature of this sample and falls beyond the scope of this manuscript.

To evaluate the changes in κ_lat_(T), we have modelled κ-LT/ρ below 500 K using the Callaway model [37,38,39]. This follows earlier work that included the TiNiSn sample and considers boundary, point-defect, and Umklapp phonon scattering [30]. The Callaway model is based on the Debye heat capacity for a monoatomic cubic lattice [37]

(4) $$\kappa_{\mathrm{lat}}=\frac{k_{B}}{2\pi^{2}\nu_{s}}{\left(\frac{k_{B}T}{\hbar }\right)}^{3}\int_{0}^{\frac{\theta_{D}}{T}}\frac{x^{4}e^{x}}{\tau_{\mathrm{ph}}^{-1}{\left(e^{x}-1\right)}^{2}}\mathrm{dx}$$

Here x is the reduced energy, k_B_ is Boltzmann’s constant, ħ is the reduced Planck constant, ν_s_ = 3630 m s^−1^ is the velocity of sound for TiNiSn [40], θ_D_ = 367 K is the Debye temperature for TiNiSn [24] and τ_ph_^−1^ is the phonon relaxation time

(5) $$\tau_{\mathrm{ph}}^{-1}=\frac{\upsilon_{s}}{D}+A_{\mathrm{PD}}\omega^{4}+B_{U}{T\omega }^{2}a$$

Here, D is the boundary scattering parameter and A_PD_ and B_U_ are the coefficients for point-defect and Umklapp scattering, respectively. A_PD_ = (V_at_/4πv_s_^3^)Γ, where Γ is the disorder parameter and V_at_ is the average volume per atom. As derived previously [30] Γ can be estimated from the mass of the interstitial atom (M_interstitial_), the average atomic mass (M_av_), and an additional −2 term to take into account bonding between the interstitials and host lattice

(6) $$\Gamma_{M}=0.25x\left(1-x\right)\left[{\left(\frac{-M_{\mathrm{interstitial}}}{M_{\mathrm{av}}}-2\right)}^{2}\right]$$

In the fits D = 5 µm and B_U_ = 2.1 × 10^−18^ s K^−1^ were used, while A_PD_ could vary freely, yielding values for Γ. The final fits are shown in Figure 5e and reproduce the temperature dependence and magnitude of κ_lat_ well. The Γ values from the fit are compared to the expected Γ_M_ from equation 6 in Table 3. This reveals an overall good agreement with the Γ_M_ equal or larger than the fitted values, demonstrating that point-defect phonon scattering can explain the observed reductions in κ_lat_. The inset to Figure 5e shows the predicted κ_lat_(T) for stoichiometric TiNiSn (i.e., Ni_4d_ = 0) based on the fitted values for D and B_U_ and obtained by setting Γ = 0.

The temperature dependence of ZT is shown in Figure 5f. ZT increases from 0.03–0.07 at 323 K to peak ZT = 0.35–0.4 at 600–750 K. These values are somewhat lower than peak ZT = 0.5–0.6 reported in the literature [17,18,19,35].

## 4. Discussion

This paper assesses the impact of interstitial Ni on the thermal and charge transport of TiNi_1+y_Sn HH alloys prepared via solid-state reactions and hot pressing. All samples are found to have a higher than expected interstitial Ni_4d_ site occupancy, signaling the difficulty in making stoichiometric TiNiSn. The lowest Ni_4d_ = 0.019 value was found for the sample with nominal Ti_1.02_NiSn composition. The spontaneous presence of excess Ni could have a thermodynamic reason, related to structural stability, or it could be due to a sluggish reaction between Ti, Ni, and Sn. The maximum Ni solubility limit is near 8% occupancy of the Ni_4d_ site. Peak shape analysis of SXRD data is consistent with partial segregation of interstitial Ni upon hot pressing, consistent with the limited solubility of interstitial Ni from phase diagram calculations.

**Figure 6:**
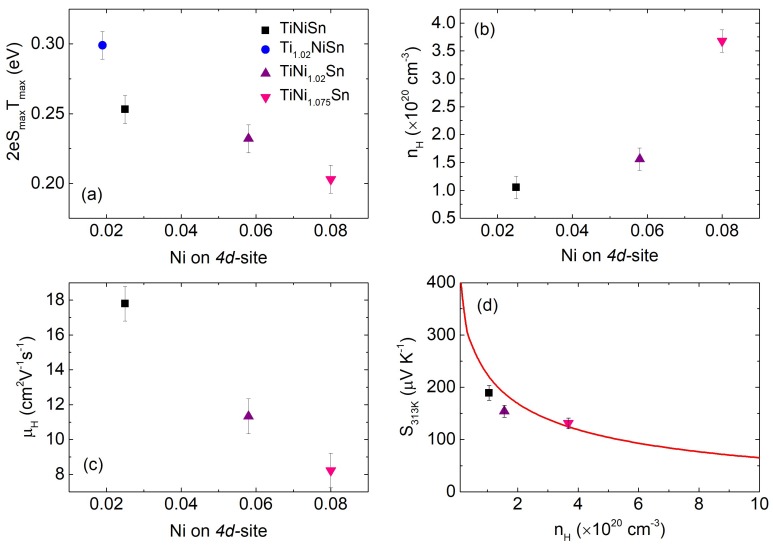
(**a**) Thermal band gap (E_g_), (**b**) Hall carrier concentration (n_H_), and (**c**) Hall mobility (µ_H_) versus Ni-4d site occupancy. (**d**) Pisarenko plot using a single Kane band with carrier effective mass m = 2.8 m_e_.

The thermoelectric property data in Figure 5 suggests that interstitial Ni leads to weak n-type doping, which maintains a semiconducting S(T) and ρ(T). The reduction of ρ(T) is offset by decreases in S(T) and the weak n-type doping does not lead to useful improvements in S^2^/ρ. This contrasts with the behaviour seen with strong n-type dopants such as Cu on the 4d-site or Sb doping on the Sn-site which lead to large improvements in S^2^/ρ [30,35,41]. As noted in the literature [23,42,43,44], electronic states due to interstitial Ni show up within the band gap of TiNiSn. A plot of the thermal band gap (E_g_), estimated from S(T) using the Goldsmid–Sharp formula [45], against Ni_4d_ site occupancy is given in Figure 6a. This reveals a gradual reduction from E_g_ = 0.3 eV for Ni_4d_ = 0.019 to E_g_ = 0.2 eV for Ni_4d_ = 0.08. To gain a better insight into the impact of interstitial Ni, Hall measurements were undertaken on the y = 0, 0.02 and 0.075 samples. The carrier concentration (n_H_) and Hall mobility (µ_H_) were extracted using a single band approximation and are shown in Figure 6b,c. n_H_ increases from 1.1 × 10^20^ cm^−3^ to 3.7 × 10^20^ cm^−3^, while µ_H_ decreases from 18 cm^2^ V^−1^ s^−1^ to 8 cm^2^ V^−1^ s^−1^ going from Ni_4d_ = 0.025 to Ni_4d_ = 0.08. The 3–4-fold increase in n_H_ is consistent with the weak n-type doping inferred from S(T) and ρ(T). The reduction in µ_H_ reveals a detrimental impact on the charge transport due to the introduction of interstitial Ni. Finally, to confirm the validity of the single band approximation, we evaluated the n_H_ dependence of S within the single Kane band (SKB) model. The SKB model has been shown to be more appropriate for materials with a small band gap than the single parabolic band model [15]. The analysis presented here follows the SKB model applied to ZrNiSn with spontaneous excess Ni and the reader is referred to [46] for further detail. The resulting Pisarenko plot is shown in Figure 6d, where an effective mass m* = N_V_^2/3^m_b_ = 2.8 m_e_ was used. Here, N_V_ = 3 is the band degeneracy, m_b_ is the band mass and m_e_ is the free electron mass. This band mass has been found to be valid for a range of ZrNiSn-based HHs [15]. Our data agree well with the predicted S-n_H_ dependence (Figure 6d). The band mass therefore does not appear to change significantly with increasing Ni content, suggesting a limited direct impact on the conduction band structure. The gradual reduction of µ_H_ = eτ/m_I_, where τ is the carrier relaxation time and m_I_ is the inertial carrier mass, is therefore likely due to a decrease in τ rather than an increase in effective mass. This decrease in τ could be caused by the introduction of interstitial Ni but may also be related to microstructure effects. We note that larger µ_H_~30 cm^2^ V^−1^ s^−1^ have been reported for TiNiSn samples where the microstructure has been optimised to be free of secondary phases [35].

As discussed, Ni interstitials cause a reduction of E_g_, which leads to weak n-type doping without affecting the magnitude of S^2^/ρ significantly. The Hall data explain this lack of improvement, which is due to the reduction of µ_H_ that offsets any improvement in ρ(T) due to the increase of n_H_. The impact of interstitial Ni on the thermal conductivity is significant with defect-free TiNiSn predicted to have κ_lat, 323K_ = 12.7 W m^−1^ K^−1^ from the Callaway modeling. This value is twice the observed κ_lat, 323 K_ = 6 W m^−1^ K^−1^ for TiNiSn with Ni_4d_ = 0.025, demonstrating that even small amounts of interstitial Ni lead to a rapid drop in κ(T). The predicted values for stoichiometric TiNiSn are in good agreement with recent phonon calculations that yield κ_lat_ = 14–15 W m^−1^ K^−1^ near room temperature [47,48]. The reduction of κ(T) is most pronounced near room temperature. The y = 0.075 sample has the lowest κ(T) of the samples investigated (Figure 5d,e). The y = 0.25 sample has higher κ(T) due to increases in k_el_ and the presence of 25 wt % metallic TiNi_2_Sn. Like the negative impact on S^2^/ρ, the presence of large amounts of metallic TiNi_2_Sn is therefore detrimental to achieving low κ(T) and should be avoided for applications. Combined with the shift of the peak S^2^/ρ towards lower temperatures due to the reduced E_g_, this leads to an almost constant peak ZT = 0.4 that can be adjusted from 600 to 800 K via the amount of interstitial Ni. This leads to the possibility to create HHs with a compositional gradient that have a broad maximum ZT plateau.

To conclude, our study confirms that interstitial Ni strongly reduces κ_lat_ but also leads to a reduction of the charge carrier mobility, limiting the attainable power factor and overall values of ZT. Interstitial Ni leads to a reduction of the thermal band gap, which in principle allows for the engineering of a broad peak ZT plateau. These results provide insight into the competing effects of interstitial Ni on charge and thermal transport and help to explain the difficulty in optimising ZT of these materials.

## Figures and Tables

**Table 1:** Half- and full-Heusler lattice parameters (a), weight percentages of the phases present and percentage density for the TiNi_1+y_Sn samples and Ti_1.02_NiSn.

Composition	Half-Heusler		Full-Heusler		Sn	Percentage Density
	a (Å)	wt %	a (Å)	wt %	wt %	
TiNiSn	5.9304(1)	97.7(1)	-	-	2.3(1)	88(2)
Ti_1.02_NiSn	5.9292(1)	95.9(1)	-	-	4.1(1)	94(2)
TiNi_1.02_Sn	5.9382(1)	93.8(1)	6.072(2)	2.1(3)	4.1(1)	95(2)
TiNi_1.075_Sn	5.9384(1)	92.8(1)	6.0745(3)	4.7(2)	2.5(1)	95(2)
TiNi_1.25_Sn	5.9391(1)	73.7(2)	6.0770(1)	26.3(2)	-	90(2)

**Table 2:** Lattice parameters (a), site occupancies (occ), thermal displacement parameters (U_iso_/Å^2^), weight percentages Sn and Ti, and fit statistics for the Rietveld fits to Polaris neutron powder diffraction data for Ti_1.02_NiSn and TiNi_1.02_Sn. Bank 3–5 are the low (2θ ~ 52°), 90° (2θ ~ 93°) and backscattering (2θ ~ 147°) banks on the Polaris instrument. wRp and Rp are defined in the GSAS manual [31].

		Ti_1.02_NiSn	TiNi_1.02_Sn
a (Å)	-	5.92951(4)	5.93607(3)
Ti (4a)	Occ	0.978(3)	0.977(2)
-	Uiso	0.00455(8)	0.00435(7)
Ni (4c)	Uiso	0.413(4)	0.00470(4)
Ni (4d)	Occ	0.019(1)	0.058(1)
-	Uiso	0.413(4)	0.00470(4)
Sn (4b)	Uiso	0.00387(6)	0.00415(5)
Wt% Sn	-	2.5(2)	3.4(2)
Wt% Ti	-	3.8(2)	4.0(2)
wRp (%)	Bank 3	2.9	2.5
-	Bank 4	2.2	1.9
-	Bank 5	2.0	1.9
Rp (%)	Bank 3	2.6	2.4
-	Bank 4	4.0	4.0
-	Bank 5	2.9	2.7

**Table 3:** Ni 4d-site occupancies, lattice thermal conductivity (κ_323K_-LT/ρ), experimental (Γ) and calculated (Γ_M_) mass disorder parameters for the TiNi_1+y_Sn samples and Ti_1.02_NiSn.

Composition	Ni 4d-Site Occupancy	κ_323K-_LT/ρ (W m^−1^ K^−1^)	Γ ^b^	Γ_M_
TiNiSn	0.025(1)	6.0	0.06	0.06
Ti_1.02_NiSn	0.019(1)	7.0	0.04	0.04
TiNi_1.02_Sn	0.058(1)	5.1	0.09	0.13
TiNi_1.075_Sn	0.08(1) ^a^	4.2	0.13	0.16
TiNi_1.25_Sn	0.09(1) ^a^	4.4	-	-

^a^ Estimated from lattice parameter; ^b^ B_U_ = 2.1 × 10^−18^ s^−1^, L = 5 µm in the Callaway fits to κ_lat_(T).
